# Refractory Coronary Vasospasms in a Patient With Eosinophilic Granulomatosis With Polyangiitis

**DOI:** 10.1016/j.jaccas.2025.104139

**Published:** 2025-07-23

**Authors:** Oumara Alajlouni, Saba Shahab, Hirmand Nouraei, Lauren Glick, Sahil Koppikar

**Affiliations:** a Department of Medicine, University of Toronto, Toronto, Ontario, Canada; b Division of Cardiology, Department of Medicine, McMaster University, Hamilton, Ontario, Canada; c Division of Cardiology, Department of Medicine, Mayo Clinic, Rochester, Minnesota, USA; d Division of Rheumatology, Department of Medicine, University of Toronto, Toronto, Ontario, Canada

**Keywords:** autoimmune, cardiac magnetic resonance, cardiac pacemaker, cardiovascular disease, coronary angiography, coronary circulation, echocardiography, fibrosis, imaging, secondary prevention

## Abstract

**Background:**

Coronary vasospasms refractory to calcium channel blockers and nitrates may be secondary to a systemic process. Although rare, eosinophilic granulomatosis with polyangiitis (EGPA) can cause coronary vasospasms.

**Case Summary:**

A 33-year-old woman presented with recurrent chest pain leading to multiple episodes of cardiac arrest. Rising eosinophilia and coronary intimal thickening were noted, and cardiac magnetic resonance imaging showed scarring. Owing to the high index of suspicion for EGPA, she was started on steroid therapy and cyclophosphamide. With this treatment, she had no further episodes of cardiac symptoms.

**Discussion:**

Cardiac involvement in EGPA is rare and carries a poor prognosis. The most common cardiac manifestations are congestive heart failure, pericarditis, myocarditis, and arrhythmias.

**Take-Home Messages:**

EGPA should be considered in the differential diagnosis for patients with refractory coronary vasospasms and eosinophilia >10%. EGPA cannot be ruled out solely based on the absence of antineutrophil cytoplasmic antibodies, particularly in patients with cardiac involvement.

## Graphical Abstract

**Figure:**
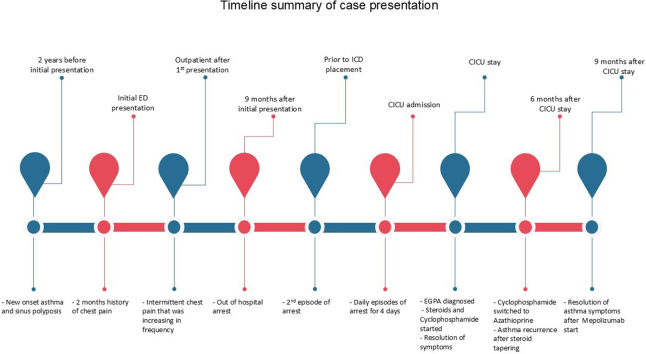

## History of Presentation

A 33-year-old woman presented with a 2-month history of chest pain. She was found to have transient anteroseptal ST-segment elevations, with visualization of coronary vasospasm during angiography and nitroglycerin responsiveness. Despite treatment with a calcium channel blocker and nitrate, over the next 9 months, she experienced 6 nitroglycerin-responsive anginal episodes, which were attributed to recurrent vasospasms, including one with syncope, leading to an out-of-hospital cardiac arrest.

**Take-Home Messages**

• EGPA should be considered in the differential diagnosis in patients who present with refractory coronary vasospasms and have eosinophilia >10%.
• EGPA cannot be definitively ruled out solely based on the absence of antineutrophil cytoplasmic antibodies, particularly in patients with cardiac involvement.

**Figure 1:**
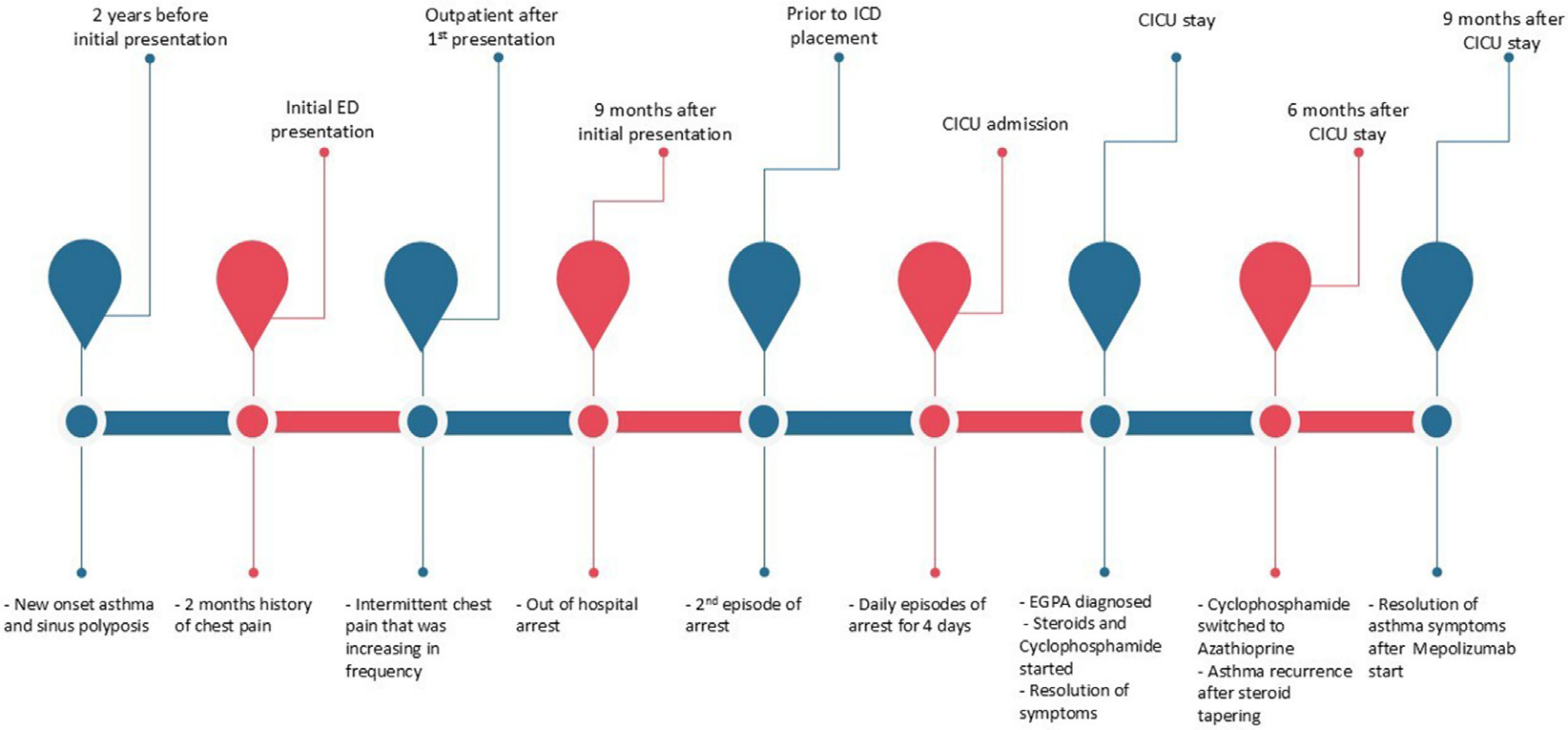
Timeline Summary of Case Presentation CICU = cardiac intensive care unit; ED = emergency department; EGPA = eosinophilic granulomatosis with polyangiitis; ICD = implantable cardioverter-defibrillator.

**Figure 2:**
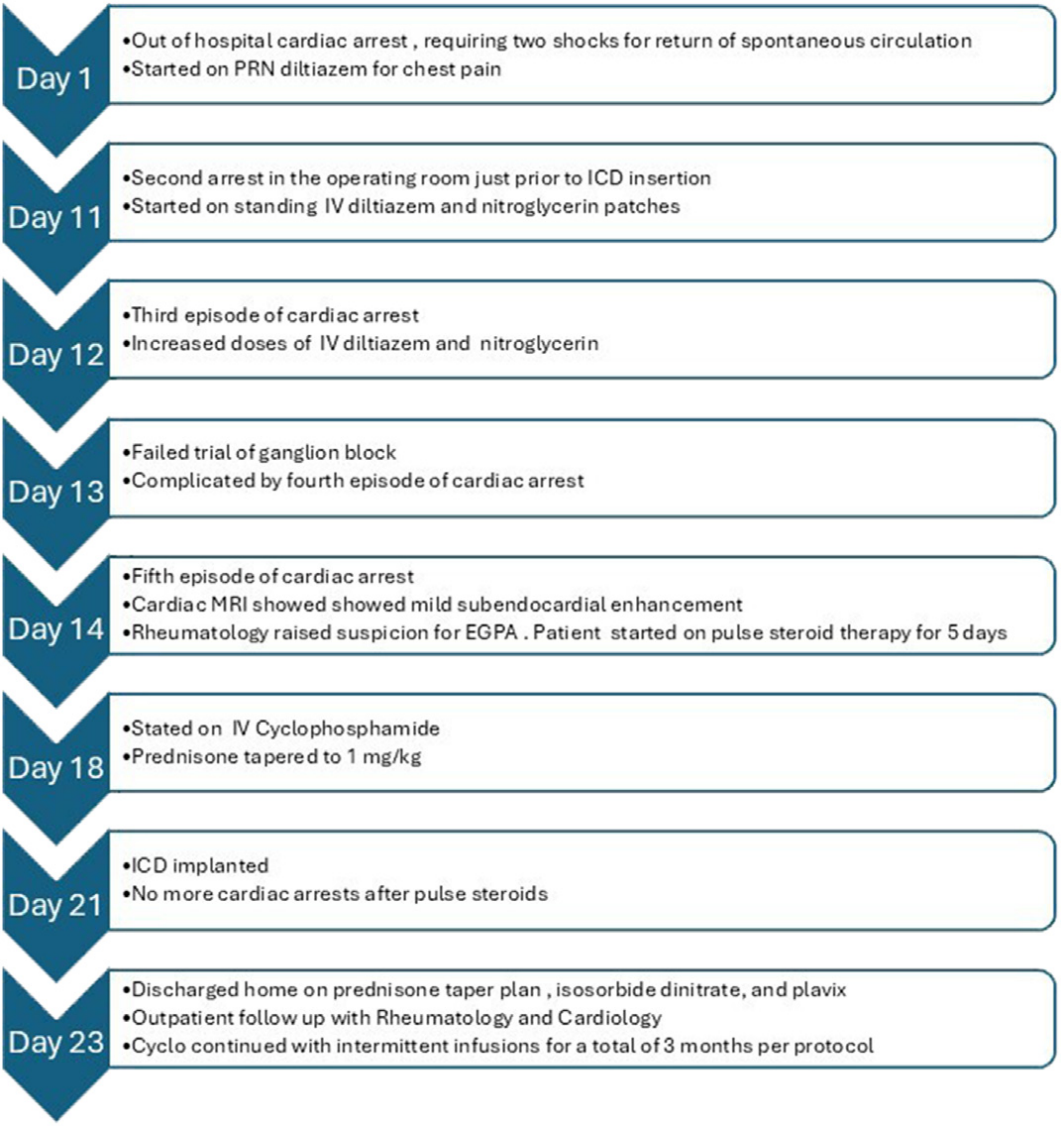
Sequence of Events in the CICU CICU = cardiac intensive care unit; EGPA = eosinophilic granulomatosis with polyangiitis; ICD = implantable cardioverter-defibrillator; IV = intravenous; MRI = magnetic resonance imaging.

The cardiac arrest was characterized by polymorphic ventricular tachycardia followed by asystole, requiring 2 shocks for return of spontaneous circulation. A subcutaneous implantable cardioverter-defibrillator (ICD) was recommended for secondary prevention. She sustained a second cardiac arrest just before ICD insertion, resulting in the procedure being aborted. The patient was transferred to the cardiac intensive care unit (CICU) where she had 3 subsequent cardiac arrests. A timeline summary of her presentation and stay in the CICU is given in Figures 1 and 2.

## Past Medical History

She had a history of adult-onset asthma, hypothyroidism, and sinus polyposis. No history of cardiovascular risk factors. She did not smoke or use any illicit drugs including cocaine.

## Differential Diagnosis

The differential diagnoses for refractory coronary vasospasms with eosinophilia include eosinophilic granulomatosis with polyangiitis (EGPA), Kounis syndrome, aspirin-exacerbated respiratory disease, hypereosinophilic syndrome, hematologic malignancies, and infections caused by parasites and fungi as they can present with similar clinical features involving the heart and eosinophilic infiltration.

## Investigations

Given the broad differential diagnosis, a peripheral flow cytometry and bone marrow biopsy, along with an extensive infectious work-up, were done to exclude these possible etiologies. Despite a positive rheumatoid factor result, there was no indicative history of rheumatoid arthritis (RA), and RA-associated vasculitis would not typically manifest in the absence of clinical RA symptoms. Test findings for immunoglobulins, antineutrophil cytoplasmic antibodies (ANCAs), complements, antinuclear antibodies, cryoglobulins, stool cultures for ova and parasites, and hepatitis serologies were negative.

Eosinophilia was noted on her bloodwork, with eosinophils increasing from 1.5 cells/L on her presentation to 6.7 cells/L in the CICU (50% of total white blood cell count), alongside elevated immunoglobulin E (920 IU/mL) and rheumatoid factor (47 U/mL). Computed tomography of the sinus confirmed polypoid mucosal thickening.

**Figure 3:**
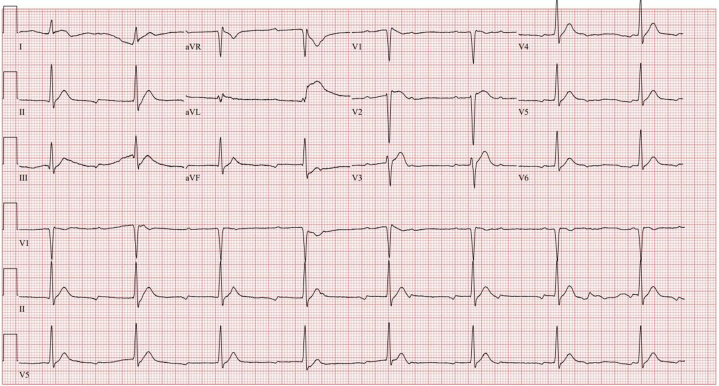
Pre-Arrest Electrocardiogram Showing Complete Heart Block

**Figure 4:**
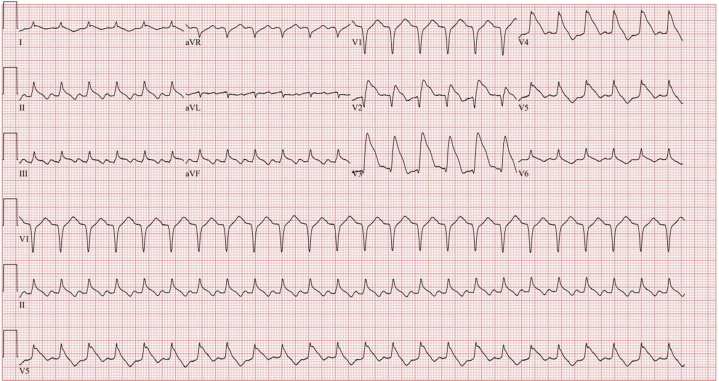
Pre-Arrest Electrocardiogram Showing ST-Segment Elevations

Initial cardiac magnetic resonance (CMR) imaging showed no fibrosis and a left ventricular ejection fraction (LVEF) of 49%. Prearrest electrocardiograms showed complete heart block and ST-segment elevations (Figures 3 and 4). Computed tomography findings of the chest, abdomen, and pelvis were normal. A transthoracic echocardiography the next day after her first cardiac arrest showed an LVEF of 50% to 55%, with no regional wall motion abnormalities. An echocardiogram after her second cardiac arrest showed an LVEF of 25%. CMR imaging after her fifth arrest showed mild subendocardial enhancement involving the midanterior and mid-to-distal anteroseptal wall.

Considering the patient's history of asthma and nasal polyposis, along with increasing eosinophilia, and late gadolinium enhancement and myocardial edema on CMR imaging, a diagnosis of EGPA was suspected by rheumatology.

## Management

She was initially treated with aspirin, diltiazem, rosuvastatin, isosorbide mononitrate, and nitroglycerin after her initial presentation. A subcutaneous ICD was recommended before the diagnosis of EGPA was made but was delayed because of several subsequent arrests. Despite treatment with maximum doses of intravenous (IV) diltiazem and nitroglycerin, she experienced 3 subsequent cardiac arrests. A left stellate ganglion block was conducted by an anesthesiologist after her third episode of cardiac arrest as second-line therapy for refractory vasospasms but was not effective.

After failure of conventional treatment, EGPA was suspected, and she was given pulse steroid therapy for 5 days, followed by prednisone 1 mg/kg/d. After ruling out infectious and hematologic etiologies of vasospasm and eosinophilia, she was started on IV cyclophosphamide for induction therapy. With this therapy, her symptoms abated, and eosinophils, C-reactive protein, and erythrocyte sedimentation rate normalized. She had an ICD inserted and was discharged home on prednisone, isosorbide dinitrate, and plavix.

## Outcome and Follow-Up

She continued to receive IV cyclophosphamide for EGPA for 3 months, alongside a steroid taper. After this, azathioprine was added for remission maintenance. With this therapy, her eosinophil count normalized, and she had no further episodes of vasospasm, arrhythmia, or cardiac arrest. On tapering prednisone, her asthma symptoms relapsed with documented reversible airway obstruction on pulmonary function tests. Mepolizumab was added, and steroids were subsequently tapered off, with no relapse of symptoms. Amlodipine, plavix, and isosorbide dinitrate were discontinued. Currently, the patient has been symptom-free for 1.5 years and is being followed by rheumatology. Follow-up at the device clinic showed no recurrent ventricular arrhythmias.

## Discussion

This case highlights the importance of investigating systemic causes for refractory coronary vasospasms. Given the etiology of underlying EGPA as this patient's cause for coronary vasospasm, the standard treatment of vasospasm proved insufficient in isolation. High-dose steroids and immunosuppressive therapy were initiated with good effect, with no further episodes of vasospasm or arrhythmia.

Coronary vasospasms can present as stable angina, acute coronary syndrome, or even sudden cardiac death. Vasospasms can be diagnosed through visualization of the spasm during coronary angiography after administration of an agent that provokes vasoconstriction.^1^ First-line treatment for coronary vasospasm includes lifestyle changes including smoking cessation and the use of calcium channel blockers and nitrates.^1^ Nonpharmacologic therapies for refractory coronary vasospasms include coronary bypass surgery, stent placement, and ICD placement for spasm-induced lethal arrhythmias.^1^ Coronary vasospasms would be considered refractory if they occur despite maximum doses of first-line pharmacologic therapies. Secondary causes of coronary vasospasms include chemotherapy agents, infectious myocarditis, emotional stress, and smoking.^1^

EGPA is an autoimmune vasculitis affecting small and medium arteries. EGPA commonly presents with chronic rhinosinusitis, asthma, and eosinophilia. It primarily impacts the lungs but can involve other organ systems, including the cardiovascular and gastrointestinal systems. Approximately 60% of EGPA cases have absent ANCA autoantibodies, and these individuals more commonly exhibit cardiac manifestations.^2^ The 2022 American College of Rheumatology/European League Against Rheumatism classification criteria for EGPA include key indicators that are summarized in Table 1.^2^ At the time of her admission, the patient did not meet the classification criteria for EGPA, as certain investigations could not be conducted while the patient was in a critical condition. However, after the index admission, we were able to document reactive airways on pulmonary function tests, nasal polyposis, and eosinophilia >1, consistent with a diagnosis of EGPA.

**Table 1:** Overview of the 2022 American College of Rheumatology/European League Against Rheumatism Classification Criteria for EGPA

Criteria	Details	Points
Blood eosinophil count	≥1×10^9^/L (high eosinophil levels)	5
Obstructive airway disease	Commonly presents as asthma	3
Presence of nasal polyps	Presence of nasal polyps	1
Extravascular eosinophilic predominant inflammation	Involvement of eosinophilic inflammation in tissues	2
Cytoplasmic ANCA or anti-proteinase 3 ANCA positivity	Negative indicator – ANCA positivity lowers likelihood of EGPA	−3
Hematuria	Negative indicator – presence of blood in urine lowers likelihood of EGPA	−1

ANCA = antineutrophil cytoplasmic antibody; EGPA = eosinophilic granulomatosis with polyangiitis.

Cardiac involvement in EGPA carries a poor prognosis and is the primary cause of death among patients with the disease. The most common cardiac manifestations are congestive heart failure, pericarditis, myocarditis, and arrhythmias.^3^ In a study by Arunachalam et al, 16% of 383 patients with EGPA had cardiomyopathy and 15% had pericarditis with endomyocardial involvement confirmed by CMR imaging.^3^ Therefore, it is critical to think of potential cardiac involvement in patients with established EGPA, and similarly, to think of EGPA in patients with atypical, refractory, or unusual cardiac presentations. In ANCA-negative individuals, eosinophilic infiltrates contribute to cardiac, gastrointestinal, and pulmonary manifestations, with less vasculitis on histopathologic analysis compared with that in ANCA-positive cases.^4^ Current literature on 4 similar cases of isolated cardiac involvement highlights the diverse clinical and cardiac manifestations of EGPA.^5^, ^6^, ^7^, ^8^ Common findings across the 4 cases include chest pain, eosinophilia, and cardiac involvement, ranging from myocarditis to coronary vasospasm and myocardial infarction. Most patients had elevated inflammatory markers, immunoglobulin E, and troponin. All had normal coronary angiography, distinguishing EGPA from atherosclerotic disease. CMR revealed cardiac abnormalities identifying inflammatory changes and delayed enhancement. Treatment with corticosteroids, combined with immunosuppressive agents (cyclophosphamide or rituximab), resulted in symptom resolution and improved cardiac function. Variable outcomes were described in these cases, ranging from persistence of focal aneurysms to complete recovery. However, our case was novel in the refractory nature of vasospasm despite maximal medical therapy, the occurrence of multiple cardiac arrests, and the use of mepolizumab after steroid tapering.

For patients with severe EGPA and life-threatening manifestations, the Canadian Vasculitis Research Network guidelines recommend including cyclophosphamide or rituximab for remission induction, with a preference for cyclophosphamide in cases of cardiac involvement.^9^ Literature supports cyclophosphamide's efficacy in EGPA, with studies revealing significantly extended survival when used alongside glucocorticoids as opposed to glucocorticoids alone.^9^

Current guidelines suggest ICD therapy in patients with coronary vasospasms who have had a cardiac arrest, with a Class IIb recommendation in patients who are responsive to medical therapy.^10^ However, there are no specific guidelines for patients with a known secondary cause for vasospasm, including EGPA-associated coronary vasospasm. In this case, on the basis of the patient's age, high risk of sudden death, and the possibility that EGPA may not be fully treated, an ICD was offered.

## Conclusions

Coronary vasospasms that are refractory to conventional treatments including risk factor modification, calcium channel blockers, and nitrates should be thoroughly investigated for secondary causes, including rare presentations of rheumatologic diseases. This can be best accomplished through a thorough history and physical examination for both cardiac and noncardiac symptoms and involvement of a multidisciplinary team.

## Funding Support and Author Disclosures

Dr Koppikar has received consulting fees, honoraria, or educational/research grants from AbbVie, Celltrion, Eli Lilly, Fresenius Kabi, JAMP, Janssen, Novartis, Pfizer, Sandoz, and UCB. All other authors have reported that they have no relationships relevant to the contents of this paper to disclose.
